# Subxiphoid thoracoscopic thymectomy for myasthenia gravis

**DOI:** 10.1093/icvts/ivab262

**Published:** 2021-10-09

**Authors:** Yuanyuan Liu, Jinghao Zhang, Wenbin Wu, Hui Zhang, Chen Zhao, Miao Zhang

**Affiliations:** 1 Department of Respiratory and Critical Care Medicine, Xuzhou Central Hospital, Xuzhou, China; 2 Department of Cardiothoracic Surgery, Xuzhou Central Hospital, Xuzhou, China

**Keywords:** Subxiphoid, Thymectomy, Myasthenia gravis

## Abstract

We investigated the efficacy of subxiphoid thoracoscopic thymectomy in patients with myasthenia gravis. The data of 37 consecutive cases were reviewed. 2 cases of postoperative myasthenia gravis crisis and 4 cases of residual mediastinal fat tissue were recorded. Moreover, 29 patients presented the neurological outcomes, and complete stable remission was achieved in 5 (17.2%) cases. Subxiphoid thymectomy is technically feasible. High-quality evidence is warranted before this approach can be recommended.

## INTRODUCTION

The treatment options for myasthenia gravis (MG) include medication and thymectomy; thymectomy plus medication provides better remission of MG and reduced risk of recurrence compared with medication alone [1]. A randomized trial proved that extended trans-sternal thymectomy provided enduring long-term benefits in patients with generalized non-thymomatous MG [2].

A subxiphoid thoracoscopic thymectomy is also technically feasible [3], which might allow an extended thymectomy that is as adequate as a thymectomy by a sternotomy [4]. But, it also raises the concern of the incomplete dissection of residual or ectopic thymic tissue in the anterior mediastinum, which might result in compromised neurological efficacy. To our knowledge, the role of a subxiphoid thymectomy in MG remains largely unknown. Therefore, we conducted a single-centre cohort study.

## PATIENTS AND METHODS

The clinical data of patients undergoing a thymectomy between February 2016 and April 2020 at the Xuzhou Central Hospital were reviewed. The Myasthenia Gravis Foundation of America (MGFA) classification of the patients was determined [5]. The patients chose the subxiphoid approach themselves. The inclusion criteria were (i) thymomatous or non-thymomatous MG; (ii) computed tomography (CT)-excluded tumour invasion to the adjacent trachea, heart, aorta or major vessels; and (iii) no history of thoracic surgery or radiotherapy. As per the intent-to-treat principle, there were no specific exclusion criteria. The Institutional Review Board and Ethics Committee of Xuzhou Central Hospital approved this study (XZXY-LJ-20150110–083).

Double-lumen intubation with the patient under general anaesthesia was mandatory in all patients. The operation included *en bloc* resection of the thymus and peripheral fatty tissue as completely as possible. A uniportal and three-port thoracoscopic thymectomy was performed as reported [3, 6], and a single transverse incision about 2.5–3.5 cm was made 1 cm below the xiphoid, with or without 2 0.5-cm extrapleural thoracic ports under the bilateral costal arches. Both mediastinal pleura were opened up to the internal thoracic vessels, with the right side first. After the operation, all patients resumed taking pyridostigmine and prednisone. The dosage and times of medication were adjusted during the follow-up period according to the instructions of neurologists. The efficacy in these patients was re-evaluated as effective [including complete stable remission (CSR) and pharmacological remission (PR)] or ineffective (including no change and worse). CSR was defined as no MG symptoms for at least 1 year without medical therapy.

## RESULTS

**Figure 1:**
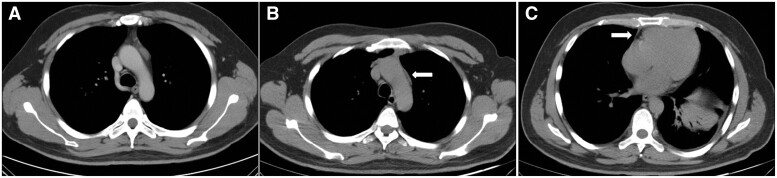
Computed tomography showed residual fat after subxiphoid thymectomy. (**A**) Thymoma was indicated before surgery; (**B**) the fat adjacent to the aortic arch; and (**C**) the soft tissue located in the cardiophrenic angle.

A total of 37 consecutive patients with MG who underwent uniportal or 3-port subxiphoid thoracoscopic thymectomy were reviewed (Table 1). Data are reported as *n* (%) or median and interquartile range. Four patients received plasmapheresis preoperatively. Massive bleeding or 90-day deaths were not recorded. Among the patients, 1 conversion to thoracotomy (2.7%), 2 cases of unilateral phrenic nerve injury (5.4%) and 2 cases of MG crisis (5.4%) were reported. No specific treatment was administrated for the 2 patients with phrenic nerve injury because they merely showed mild abdominal distention, which was relieved gradually in a year. For the patients with MG crisis, a timely plasmapheresis was effective in both patients. Specifically, the CT images revealed residual mediastinal fat tissue in 4 cases (10.8%). Among these 4 patients, 1 patient suffered from postoperative MG crisis, and the residual mediastinal fatty tissues were obviously visible in the CT images (Fig. 1).

**Table 1:** Clinical features of patients and postoperative neurological efficacy

Gender (female/male)	15/22
Age (years), median (range)	50.0 (45.0–60.0)
Body mass index (kg/m^2^), median (range)	23.9 (20.7–25.5)
MGFA classification (I/II/III/IV)	12/22/3/0
Maximal tumour diameter (cm), median (range)	2.2 (1.8–2.7)
Pathological diagnosis (thymoma/non-thymoma)	18/19
FEV_1_ (% of predicted), median (range)	65.0 (62.0–67.0)
Preoperative plasmapheresis (cases)	4
Conversion to thoracotomy/sternotomy, *n* (%)	1 (2.7)
Operation time (min), median (range)	80.0 (60.0–100.0)
Estimated blood loss (ml), median (range)	50.0 (50.0–100.0)
Myasthenia crisis after surgery, *n* (%)	2 (5.4)
Chest tube duration (days), median (range)	3.0 (2.5–4.5)
Total drainage volume (ml), median (range)	170.0 (100.0–250.0)
Hospital stay after surgery (days), median (range)	6.0 (5.0–7.5)
Wound infection/delayed healing, *n* (%)	1 (2.7)
Temporary phrenic nerve paresis, *n* (%)	2 (5.4)
Obvious residual soft tissue in anterior mediastinum by CT, *n* (%)	4 (10.8)
Patients followed up, *n* (%)	29 (78.4)
Follow-up (months), median (range)	34.0 (23.5–50.0)
Neurological outcomes, *n* (%)	
Overall clinical improvement (CSR/PR)	18 (62.1)
CSR	5 (17.2)
Ineffective (unchanged/worse)	11 (10/1)

Data are reported as *n* (%) or median and interquartile range.

CSR: complete stable remission; FEV_1_: forced expiratory volume in the first second; MGFA: Myasthenia Gravis Foundation of America; PR: pharmacological remission.

Moreover, 29 of the 37 cases (78.4%) finished the median follow-up of 34.0 (interquartile range, 23.5–50.0) months. In total, CSR and pharmacological remissions were achieved in 5 (17.2%) and 13 (44.8%) patients, respectively.

## DISCUSSION

Taioli *et al.* reviewed the neurological effect of surgical treatment for myasthenia patients, and they reported that the pooled proportion of MG remissions with thymectomy was 0.31 (95% confidence interval, 0.25–0.37) [7]. Subxiphoid thymectomy can achieve similar or better outcomes compared to the lateral approach [8], but the long-term neurological efficacy of the subxiphoid approach for MG has not been fully elucidated. Our study merely suggested the technical feasibility and safety of subxiphoid thoracoscopic thymectomy, which provided a truly limited chance of CSR (17.2%). However, our findings should be interpreted with caution because of the entirely retrospective nature, significant heterogeneity, inherent bias and limited sample size. Many questions remain unanswered.

The first issue is the safety, which underlines the importance of finishing a learning curve for the subxiphoid approach. The rate of phrenic nerve injuries in our cohort was 5.4%. Two patients suffered from MG crisis after thymectomy, and 1 patient showed significant residual fat in the mediastinum in CT images. Among these 4 patients with residual fat tissue, only 1 achieved pharmacological remission during the follow-up period. Therefore, a standard subxiphoid procedure should be established for safety concerns; meanwhile, adequate perioperative interventions should be considered for selected patients to diminish the risk of post-thymectomy MG crisis.

The second issue is the radicality of the subxiphoid approach for extended thymectomy. The impact of ectopic thymic foci on the neurological outcomes is unclear, leading to divergent views, i.e. from maximal resection of all fatty tissue (between bilateral pleura, thyroid grand, pericardium and diaphragm) to less extensive approaches. However, it is presumed that a post-thymectomy myasthenia crisis might be ascribed to the residual or ectopic thymic tissue. A review showed that the common locations of ectopic thymic tissue in mediastinal fat included anterior mediastinal fat (33.2%), pericardiophrenic angles (13.6%) and an aortopulmonary window (10.4%) [9]. Some anatomical sites (such as the pericardia-phrenic angle) might be difficult to access under the subxiphoid approach. Zielinski *et al.* [10] suggested that the complete 5-year remission rate of transcervical-subxiphoid-thoracoscopic ‘maximal’ thymectomy was 53.1%, which was statistically better than the basic trans-sternal operation. In theory, the difference could be explained by the complete removal of residual/ectopic thymic tissue in the neck and mediastinum. Further high-quality studies may provide better evidence regarding the equivalence of different thoracoscopic approaches. It is noteworthy that additional transcervical/intercostal incisions should be considered when extended thymectomy could not be guaranteed using the subxiphoid approach.

## CONCLUSIONS

Subxiphoid thoracoscopic thymectomy is technically feasible. However, considering the unsatisfactory neurological outcomes, high-quality trials are warranted before this approach can be recommended for patients with MG as a less invasive alternative for trans-sternal thymectomy.

##  

**Conflict of interest**: none declared.

### Data availability statement

The data are available from the corresponding author upon receipt of a reasonable request.

### Reviewer information

Interactive CardioVascular and Thoracic Surgery thanks Lucio Cagini, Nuria M Novoa and the other, anonymous reviewer(s) for their contribution to the peer review process of this article.
